# In Silico Evaluation and In Vitro Determination of Neuroprotective and MAO-B Inhibitory Effects of Pyrrole-Based Hydrazones: A Therapeutic Approach to Parkinson’s Disease

**DOI:** 10.3390/molecules27238485

**Published:** 2022-12-02

**Authors:** Magdalena Kondeva-Burdina, Emilio Mateev, Borislav Angelov, Virginia Tzankova, Maya Georgieva

**Affiliations:** 1 Laboratory of Drug Metabolism and Drug Toxicity, Department of Pharmacology, Pharmacotherapy and Toxicology, Faculty of Pharmacy, Medical University of Sofia, 2 Dunav Street, 1000 Sofia, Bulgaria; 2 Department of Pharmaceutical Chemistry, Faculty of Pharmacy, Medical University of Sofia, 2 Dunav Street, 1000 Sofia, Bulgaria

**Keywords:** pyrrole, hydrazones, neurotoxicity and neuroprotection, MAO-B, Parkinson

## Abstract

Parkinson’s disease is a huge burden in modern medicinal practice. A serious drawback of current antiparkinsonian therapy is its symptomatic nature. This directed our investigations in the search for new more potent derivatives, affecting not only the loss of dopaminergic neurons but also the oxidative damage of neuronal cells. Thus in vitro neurotoxicity and neuroprotective analysis on a group of *N*-pyrrolyl hydrazide–hydrazones were performed. The neurotoxicity of the target derivatives was determined on a subcellular level in isolated rat synaptosomes, mitochondria and microsomes determining their effect on cellular vitality, GSH depletion and MDA production. The neuroprotective effects of the evaluated hydrazones were measured in three models of induced oxidative stress: 6-OHDA, *t*-BuOOH and Fe^2+^/AA-induced lipid peroxidation. Molecular docking simulations along with in vitro evaluation of MAO-B inhibitory potential of the target molecules were also performed. The results identified the ethyl 5-(4-bromophenyl)-1-(3-hydrazinyl-3-oxopropyl)-2-methyl-1*H*-pyrrole-3-carboxylate (**12**) as the most promising compound with the lowest neurotoxicity and highest neuroprotection on all evaluated parameters and inhibiting the *h*MAOB enzyme by 50%, comparable with the activity of the reference, Selegiline. The compatibility of the in silico and in vitro evaluations is a good prerequisite for these methods to be applied in future assessment of pyrrole-based compounds as anti-Parkinson agents.

## 1. Introduction

Parkinson’s disease (PD) is considered the second most common neurodegenerative disorder [1] that affects approximately 1% of the world population older than 65 years, representing up to two-thirds of all patients with movement disorders throughout the world [2]. PD has become increasingly more common with advances in age, reaching the proportions of 2.6% of the population over 85 years old [3]. In addition, some data suggest that by 2020, motor development disorders will be developed worldwide as secondary to PD [3,4,5].

During the pathogenesis of PD, the production of oxygen-reactive species damages the *substantia nigra* through lipid peroxidation, protein oxidation, and DNA oxidation. This phenomenon seems to be induced mainly by changes in the iron content of the brain, mitochondrial dysfunction, monoamine oxidase (MAO) activation, or even by changes in the antioxidant defense system [3].

Much evidence still indicates that exposure to dopaminergic neurotoxins may trigger PD-related pathology. One of the ways is the effectivity of the oxidizing astrocytic monoamine oxidase type B [6,7]. Another factor is the multiple detrimental oxidative reactions [8] along with the possibility of the formation of reactive oxygen species, including superoxides (O_2_^−^) in cellular mitochondria and the additional mitochondrial dysfunction and fragmentation along with the oxidative neuronal damage [3,9]. In addition, it should also be considered that the dopaminergic neurons are extremely susceptible to oxidative stress due to the various oxidants formed from the oxidative changes caused to dopamine after its release from the synaptic vesicles, including H_2_O_2_, O_2_^−^ and hydroxyl (OH) and DA-semiquinone radicals’ formation [10].

In healthy dopaminergic neurons, levels of ROS are strictly controlled by various antioxidative mechanisms involving glutathione (GSH), superoxide dismutase (SOD) and DJ-1. These processes, however, tend to fail in patients with PD [11].

Unfortunately, the current treatments for PD do not decrease the extent of neurodegeneration, which determines the necessity of the development of new compounds and approaches to novel therapies [9]. Thus, proper agents decreasing the radical’s formation and/or affecting the uncontrolled monoamine oxidase type B effectivity would be a promising strategy in the search for new antiparkinsonian agents.

Monoamine oxidase B (MAO-B) has been believed to mediate the degradation of monoamine neurotransmitters such as dopamine. Recently this traditional belief has been challenged by demonstrating that it is not MAO-B but monoamine oxidase A (MAO-A) which mediates dopamine degradation. The actual role of MAO-B is to mediate the aberrant synthesis of GABA and hydrogen peroxide (H_2_O_2_) in reactive astrocytes of Parkinson’s disease (PD). Astrocytic GABA tonically suppresses the dopaminergic neuronal activity, whereas H_2_O_2_ aggravates astrocytic reactivity and dopaminergic neuronal death. Recently discovered reversible MAO-B inhibitors reduce reactive astrogliosis and restore dopaminergic neuronal activity to alleviate PD symptoms in rodents [12].

Often the classical symptomatic PD therapy includes monoamine oxidase B inhibitors (MAO-B). MAO-B inhibitor monotherapy has been shown to be effective and safe for the treatment of early-stage PD, while MAO-B inhibitors as adjuvant drugs have been widely applied for the treatment of the advanced stages of the illness. MAO-B inhibitors can effectively improve patients’ motor and non-motor symptoms, reduce “OFF” time, and may potentially prevent/delay disease progression [13]. Pyrrole is a five-membered heterocyclic ring template with multiple pharmacophores allowing its participation in a library of enormous lead molecules [14].

In the last several decades, interest in pyrrole and pyrrolopyrimidine derivatives has increased owing to their biological importance, such as anti-tumor, anti-microbial, anti-inflammatory, anti-diabetic, anti-histaminic, anti-malarial, anti-Parkinson, anti-oxidant and anti-viral effects, especially recently against COVID-19. These tremendous biological features have motivated scientists to discover more pyrrole and fused pyrrole derivatives, owing to the great importance of the pyrrole nucleus as a pharmacophore in many drugs [15]. In addition, some studies performed in our laboratory identified pyrrole hydrazones as promising neuroprotective agents and good MAO-B inhibitors [16].

These findings and the current study on the influence of MAO-B and oxidative stress on PD determined the aim of this research, pointed to in silico evaluation and in vitro determination of the effects of series *N*-pyrrolyl hydrazide–hydrazones as potential neuroprotective agents and MAO-B inhibitors as possible combined effects included in the PD treatment.

## 2. Results

**Figure 1:**
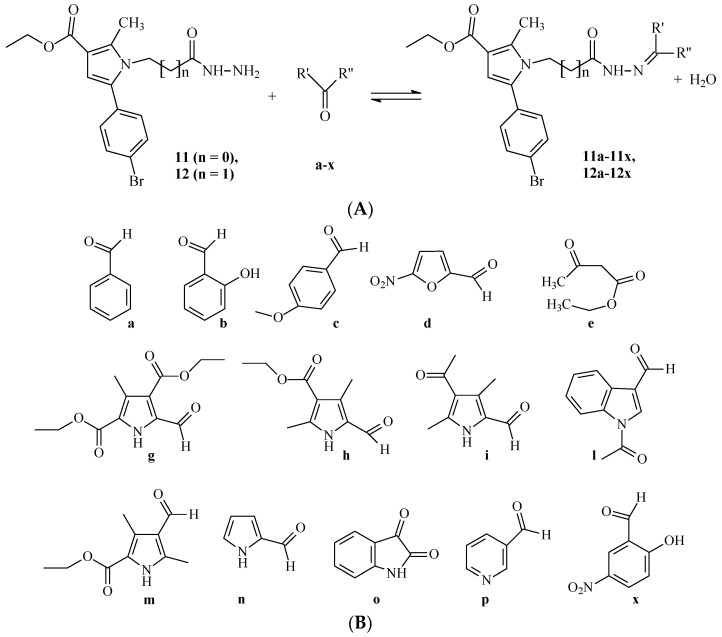
General structures of the compounds **11** and **12** and synthesis of hydrazones **11a**–**11x** and **12a**–**12x** (**A**), and structures of the corresponding carbonyl partners (**a**–**x**) (**B**).

Attempting to establish and evaluate the influence of structural parameters on neurotoxicity, neuroprotection and enzyme inhibitory activity of pyrrole-based hydrazones, the 30 compounds were selected, based on the structural variations in the methylene bridge in the hydrazide part of the structure, defining the structural difference in the hydrazones **11a**–**11x** of hydrazide **11** (ethyl 5-(4-bromophenyl)-1-(2-hydrazinyl-2-oxoethyl)-2-methyl-1*H*-pyrrole-3-carboxylate), and hydrazones **12a**–**12x** of hydrazide **12** (ethyl 5-(4-bromophenyl)-1-(3-hydrazinyl-3-oxopropyl)-2-methyl-1*H*-pyrrole-3-carboxylate) (Figure 1).

### 2.1. Molecular Docking

The dataset of the pyrrole-based hydrazide–hydrazones was docked into the active site of MAO-B (PDB ID: 1S3B) with GOLD 5.3. The most prominent compound after the utilization of the scoring function ChemPLP was **11** (134.40 PLPscore), followed by **12** (132.97 PLPscore) and **11c** (129.15 PLPscore). The MM/GBSA recalculations provided more reliable results for further testing. The top three ranks were occupied by **12a** (−56.21 kcal/mol), **12** (−54.46 kcal/mol) and **11c** (−46.44 kcal/mol), and the results are presented in Table 1.

**Figure 2:**
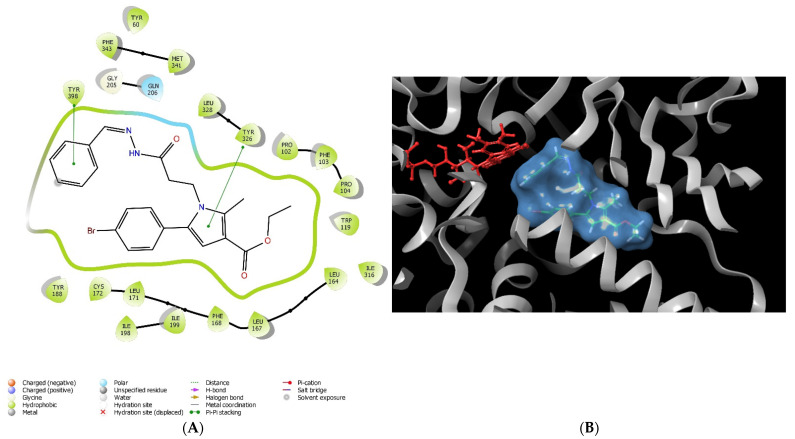
Major molecular interactions between **12a** and the active site of MAO-B (PDB: **1S3B**). The interactions are provided in 2D (**A**) and 3D (**B**) forms. The MAO-B enzyme is depicted in grey, the FAD coenzyme is colored in red, and the MAO-B inhibitor **12a** is presented in blue mesh.

Further investigations of the stabilizing intermolecular forces between the most perspective according to the docking results pyrrole-based compound (**12a**) and the active site of MAO-B were carried out (Figure 2).

Most of the stabilizing forces were hydrophobic in nature (Table 2). Moreover, the active amino acid Try326 was involved in a π−π stacking interaction with the pyrrole moiety. Gln206 was in close proximity to the hydrazide fragment and polar interactions were formed. The aromatic unsubstituted benzene ring was situated in the aromatic cage of the active site of MAO-B.

**Figure 3:**
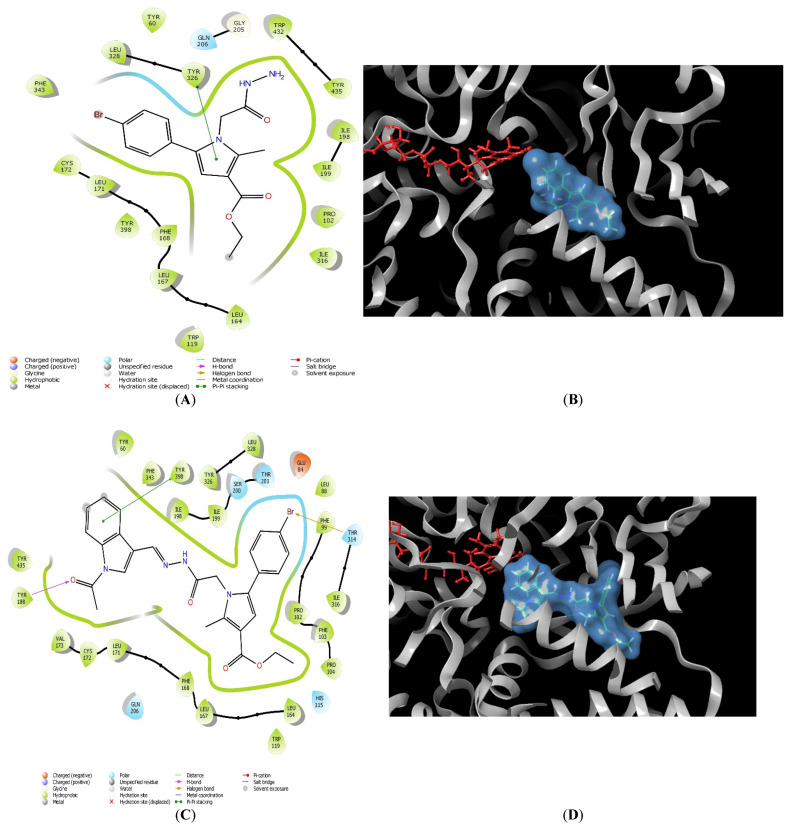

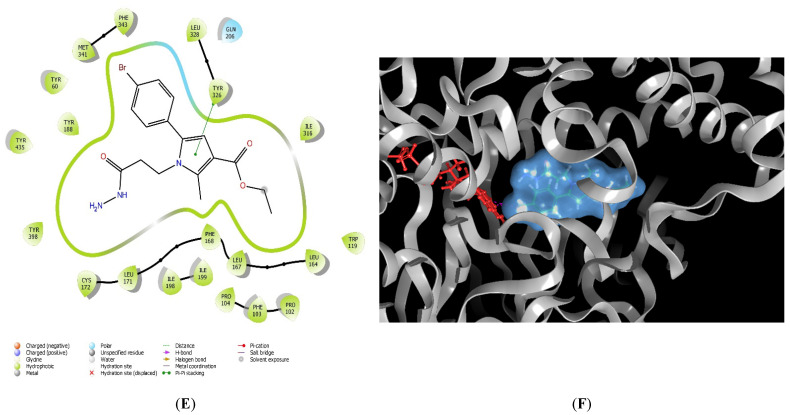
Major molecular interactions between **11**, **11l** and **12** with the active site of MAO-B (PDB: **1S3B**). The interactions are provided in 2D ((**A**,**C**,**E**), respectively) and 3D ((**B**,**D**,**F**), respectively) forms for each compound. The MAO-B enzyme is depicted in gray, the FAD coenzyme is colored in red, and the MAO-B inhibitors **11**, **11l** and **12** are presented in blue mesh.

To compare the different active conformations of the most active pyrrole-based compounds, further visualizations of **11**, **11l** and **12** in the active site of MAO-B were conducted (Figure 3). The active inhibitors **11** and **12** were stabilized by the amino residue Tyr326 (Table 2). In both cases, the active amino acid formed a stable π−π bond with the pyrrole ring. Interestingly, the active conformations of the unsubstituted hydrazides **11** and **12** were dissimilar (Figure 3A,B,E,F). Importantly, the bulky nature of compound **11l** led to several steric crashes in the active site of MAO-B, and consequently unfavorable docking score (Table 2). The indole fragment of the molecule was facing the aromatic cage, while the substituted pyrrole core was situated in the substrate cavity (Figure 3C,D).

### 2.2. In Vitro Neurotoxicity Evaluations

#### 2.2.1. Effects of the Tested Compounds **11**, **11a**–**11x**, **12**, and **12a**–**12x** on the Synaptosomal Viability of Isolated Rat Brain Synaptosomes

**Figure 4:**
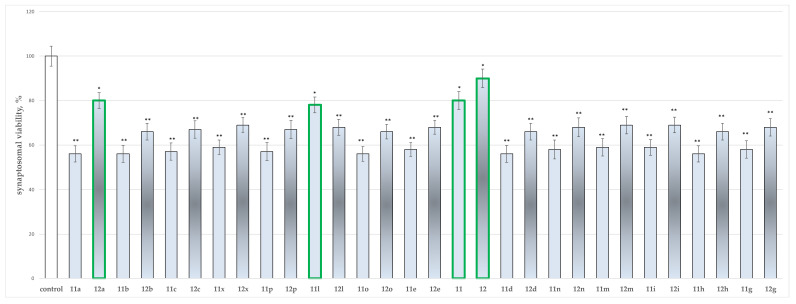
Effects of compounds **11**, **11a**–**11x**, **12** and **12a**–**12x** (100 µmol), applied alone, on synaptosomal viability. * *p* < 0.05; ** *p* < 0.01 against the control (untreated synaptosomes). The green outline indicates the most promising derivatives.

The performed screening identified the influence of a single introduction of compounds **11**, **11a**–**11x**, **12** and **12a**–**12x** in 100 µmol concentration on the cellular vitality of synaptosomal subcellular fraction of rat brain isolates. The tested synaptosomes were extracted through size or density-based fractionation of the homogenate. The obtained results are presented in Figure 4.

The results identified as least toxic, according to this parameter, compounds **12a**, **11l**, **11** and **12** with **12a** reducing the synaptosomal viability by 20%, **11l** by 22%, **11** by 20% and **12** by 10%, when compared to the control. This indicates compound **12** is the least toxic on synaptosomal viability, maintaining cellular vitality by 90%.

#### 2.2.2. Effects of the Tested Compounds **11**, **11a**–**11x**, **12** and **12a**–**12x** on the GSH Level in Isolated Rat Brain Synaptosomes

**Figure 5:**
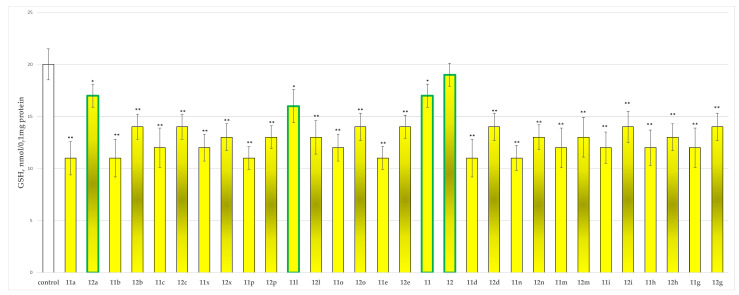
Effects of compounds **11**, **11a**–**11x**, **12** and **12a**–**12x** (100 µmol), applied alone, on the reduced GSH levels. * *p* < 0.05; ** *p* < 0.01 against the control (untreated synaptosomes). The green outline indicates the most promising derivatives.

The glutathione level is a well-known parameter for the evaluation of xenobiotics neurotoxicity. The tested compounds were incubated at 100 µmol with the isolated synaptosomal subcellular fraction. The results of their activity are presented in Figure 5.

The obtained values determined as least toxic on this parameter again compounds **12a**, **11l** and **11** with **12a** decreasing the GSH level by 15%, **11l** by 20% and **11** by 15%. Compound **12** is defined as the least toxic since it does not affect the GSH levels, preserving the GSH level comparable to the one of the control.

#### 2.2.3. Effects of the Tested Compounds **11**, **11a**–**11x**, **12** and **12a**–**12x** on the MDA Production and GSH Level in Isolated Rat Brain Mitochondria

**Figure 6:**
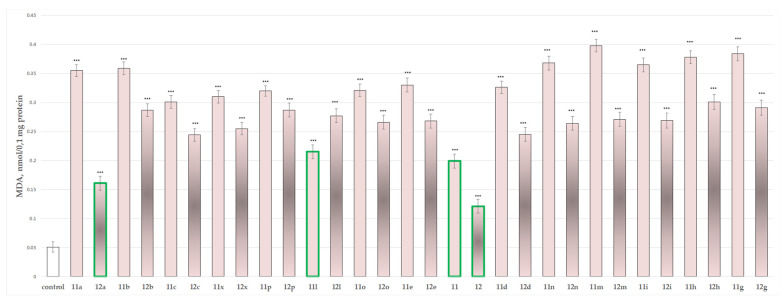
Effects of compounds **11**, **11a**–**11x**, **12** and **12a**–**12x** (100 µmol), applied alone, on MDA production. *** *p* < 0.001 against the control (untreated mitochondria). The green outline indicates the most promising derivatives.

**Figure 7:**
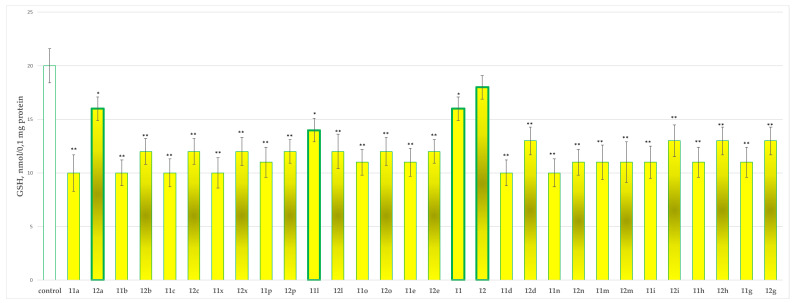
Effects of compounds **11**, **11a**–**11x**, **12** and **12a**–**12x** (100 µmol), applied alone, on the GSH levels. * *p* < 0.05; ** *p* < 0.01 against the control (untreated mitochondria). The green outline indicates the most promising derivatives.

Figure 6 and Figure 7 show the results from the incubation of the tested hydrazones **11**, **11a**–**11x**, **12** and **12a**–**12x** in the concentration of 100 µmol alone in isolated rat brain mitochondria on the MDA production and GSH levels, respectively.

On MDA production, administered alone, most of the substances showed prominent neurotoxicity, except for substances **11, 12, 11l**, and **12a** which showed weak toxicity elevating the MDA production by 290%, 137%, 320% and 216%, respectively.

On the other evaluated parameter (GSH level), **12** did not perform any statistically significant neurotoxic effect. Compounds **11**, **11l** and **12a** decreased the GSH production by 20%, 30% and 20%, respectively, against the control (untreated mitochondria).

#### 2.2.4. Effects of the Tested Compounds **11**, **11a**–**11x**, **12** and **12a**–**12x** on the Isolated Rat Brain Microsomes

**Figure 8:**
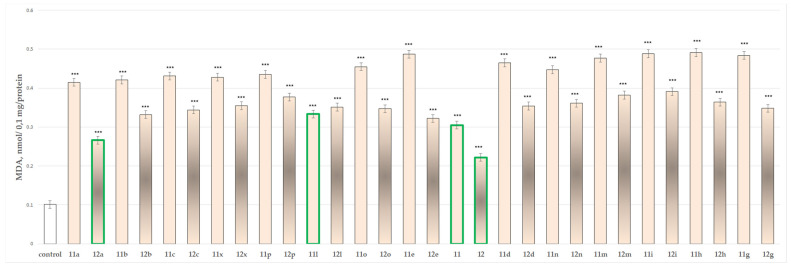
Effects of compounds **11**, **11a**–**11x**, **12** and **12a**–**12x** (100 µmol), applied alone, on MDA production in isolated rat brain microsomes. *** *p* < 0.001 against the control (untreated microsomes). The green outline indicates the most promising derivatives.

When tested alone in a concentration of 100 µmol, the evaluated *N*-pyrrolyl hydrazide–hydrazones performed pronounced toxicity on isolated rat brain microsomes, except for compounds **11**, **12**, **11l** and **12a**, which showed the lowest neurotoxicity, when compared to the control (untreated microsomes) (Figure 8).

The results indicated that the most promising derivatives are **11**, affecting the MDA production by 202%, **12** by 120%, **11l** by 230% and **12a** by 163%.

The neurotoxicity evaluations on three subcellular fractions underlined from all 30 evaluated hydrazones of two *N*-pyrrolyl hydrazides molecules **11**, **12**, **11l** and **12a** as the least neurotoxic derivatives, which were further subjected to additional evaluation of the possible neuroprotective effects.

### 2.3. In Vitro Evaluations of Neuroprotective Effects of the Tested N-Pyrrolyl Hydrazide–Hydrazones

The least toxic representatives were subjected to assessment of the possible neuroprotective effects on three models of non-enzymatic induced oxidative stress: 6-OHDA induced oxidative stress, *tert*-butyl hydroperoxide (*t*-BuOOH) induced oxidative stress, and non-enzymatic induced lipid peroxidation.

#### 2.3.1. Protective Effects of the Tested Compounds **11**, **11a**–**11x**, **12** and **12a**–**12x** in a Model of 6-OHDA-Induced Oxidative Stress of Isolated Rat Brain Synaptosomes

Two parameters were evaluated through this assessment the synaptosomal viability and the effects on the reduced GSH levels.

**Figure 9:**
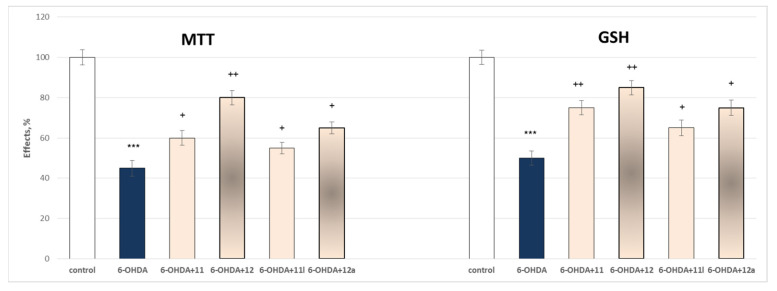
Effects of **11**, **11l**, **12** and **12a** in a model of 6-OHDA-induced oxidative stress on synaptosomal viability. *** *p* < 0.001 against the control (untreated synaptosomes),^+^ *p* < 0.05; ^++^ *p* < 0.01 against 6-OHDA.

Administered alone, 6-OHDA (150 µmol) exhibited a prominent, statistically significant neurotoxic effect relative to control (untreated synaptosomes). It reduced synaptosomal viability by 55% and GSH level by 50% (Figure 9).

The results presented in Figure 8 showed that on both parameters compound **12** exerts the most underlined statistically significant neuroprotective effect by preserving the synaptosomal viability by 78% and the GSH level by 70% against the toxic agent, followed by **12a** storing the synaptosomal viability by 44% and the GSH level by 50%, against the toxic agent. The other two evaluated compounds **11** and **11l** perform lower statistically significant neuroprotection against the toxic agent by preserving the synaptosomal viability by 33% and 22%, respectively, and storing the GSH production by 50% and 30% correspondingly for **11** and **11l**.

#### 2.3.2. Protective Effects of the Tested Compounds **11**, **11a**–**11x**, **12** and **12a**–**12x** in a Model of *Tert*-Butyl Hydroperoxide (*t*-BuOOH) Induced Oxidative Stress of Isolated Rat Brain Mitochondria

**Figure 10:**
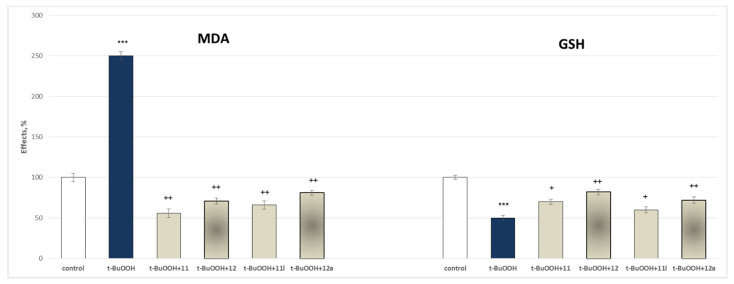
Effects of **11**, **11l**, **12** and **12a** in a model of *t*-BuOOH-induced oxidative stress on MDA production and GSH levels in isolated rat brain mitochondria. *** *p* < 0.001 against the control (untreated mitochondria),^+^ *p* < 0.05; ^++^ *p* < 0.01 against *t*-BuOOH.

Figure 10 presents the results of the evaluation of the effects of the tested hydrazide–hydrazones **11**, **12**, **11l** and **12a** in a concentration of 100 µmol on MDA production and GSH levels, used as quantitative parameters to measure the protective effects of the compounds in isolated rat brain mitochondria.

In this model, self-administered *t*-BuOOH (75 µmol) showed a pronounced statistically significant neurotoxic effect by increasing the MDA production by 150% and decreasing the GSH level by 50%, against the control (untreated mitochondria).

The figure shows that **11** decreases the production of MDA by 78%, **11l** by 74%, **12** by 72% and **12a** by 68%, against the toxic agent. On the other parameter, **11** preserves the GSH level by 40%, **11l** by 20%, **12** by 64% and **12a** by 44%, against the toxic agent. The results confirm that all substances have statistically significant neuroprotective effects with **11** affecting to a higher extent the MDA production, and **12** the GSH levels (Figure 9).

#### 2.3.3. Protective Effects of the Tested Compounds **11**, **11a**–**11x**, **12** and **12a**–**12x** in a Model of Iron Ascorbate (Fe^2+^/AA) Induced Lipid Peroxidation in Isolated Rat Brain Microsomes

**Figure 11:**
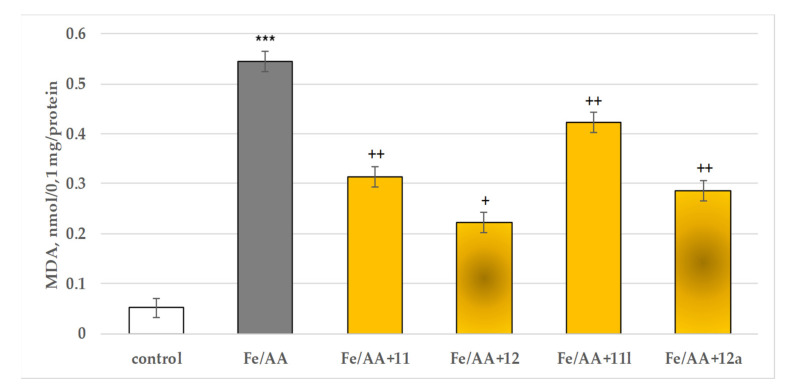
Effects of **11**, **11l, 12** and **12a** in a model of non-enzymatic induced lipid peroxidation on MDA production in isolated rat brain microsomes. *** *p* < 0.001 against the control (untreated microsomes), ^+^ *p* < 0.05; ^++^ *p* < 0.01 against Fe^2+^/AA.

When applied alone, FeSO_4_ (40 µM) together with ascorbic acid (500 µM) exhibited a pronounced statistically significant pro-oxidant effect in isolated rat brain microsomes, compared to the control (untreated microsomes) increasing the MDA production by 967% (Figure 11).

In this model, **11** reduced the MDA production by 42%, **11l** by 22%, **12** by 59% and **12a** by 47%, compared to Fe^2+^/AA (Figure 11).

### 2.4. In Vitro Evaluation of the Effects of the Tested **11**, **11a**–**11x**, **12** and **12a**–**12x** on the Activity of Human Recombinant MAO-B (hMAOB) Enzyme

An in vitro evaluation of the MAO-B inhibitory effects of the evaluated compounds was performed in an attempt to confirm the in silico identification of the expected activity of the tested series.

**Figure 12:**
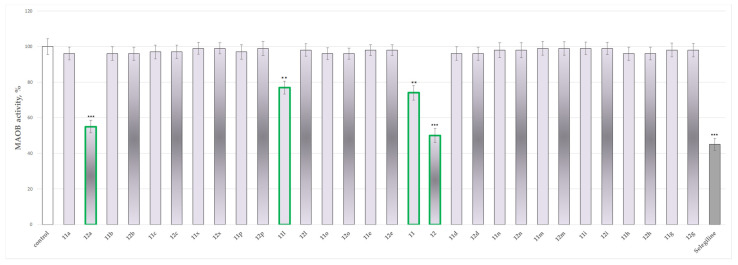
Effects of compounds **11**, **11a**–**11x**, **12**, **12a**–**12x** and Selegiline in 1 µmol concentration, applied alone, on the *h*MAOB activity. ** *p* < 0.01, *** *p* < 0.001 against the control (pure *h*MAOB).

The results presented in Figure 12 indicate that most of the tested compounds do not perform statistically significant inhibitory effects on the activity of recombinant human MAO-B. Only compounds **11**, **11l**, **12** and **12a** show low inhibitory effects by decreasing the *h*MAOB activity by 26%, 23%, 50% and 45%, respectively, against the control (pure *h*MAOB). The classical MAO-B inhibitor Selegiline was found to inhibit the enzyme by 55%.

These results bring up compound **12** as the most promising, showing inhibitory activity close to the one of Selegiline.

## 3. Discussion

### 3.1. In Silico Molecular Docking Assessment

An initial docking assessment was carried out toward the crystallographic MAO-B structure 1S3B. The optimal GOLD 5.3 scoring algorithm, grid space, as well as the RMSD value of the re-docking protocol, was previously reported by our group [17].

Interestingly, the hydrazide–hydrazone **11l** (condensed with an indole heterocycle) demonstrated a poor docking score (ChemPLP = 96.88 and MM/GBSA = 57.59), whereas the later-performed in vitro results displayed moderate MAO-B blocking activity of 23% at concentrations of 1 μM. The former docking score could be explained by the bulk structure of **11l**. Importantly, several false-positive results were present. The inactive MAO-B inhibitors **11c**, **11d** and **11n** acquired MM/GBSA scores of −46.44, −41.04 and −33.95, respectively. These results could be related to the limitations of the MM/GBSA method [18]. Overall, the obtained scores confirmed the main drawback of the in silico calculations being at risk of an elevated number of false-positive ligands in the top-scored ranks [19].

The visualization of the active conformations of the title compounds demonstrated that the active amino acid Try326 was involved in a π−π stacking interaction with the pyrrole ring. The discussed amino acid is essential for stabilization, considering it separates the entrance from the substrate cavity [20]. The most active compound **12a** formed an additional π−π bond with the active amino residue Tyr398. Furthermore, the π−π bond was formed between the phenyl moiety and the active amino residue Tyr398. Numerous hydrophobic interactions between the hydrazide and the active amino acids of MAO-B (Tyr60, Pro102, Phe103, Pro104, Trp119, Leu164, Leu167, Phe168, Leu171, Ile198, Ile199, Ile316, Phe343, Tyr398 and Tyr435) stabilized the complex. Gln206 was in close proximity to the hydrazide fragment and polar interactions were formed, which are important for enhanced activity. In addition, it was noticed that the aromatic unsubstituted benzene ring was situated in the aromatic cage of the active site of MAO-B. [21].

To compare the different active conformations of the most active pyrrole-based compounds, further visualizations of **11**, **11l** and **12** in the active site of MAO-B were conducted (Figure 3). The active inhibitors **11** and **12** were stabilized by the amino residue Tyr326. In both cases, Tyr326 formed a stable π−π bond with the pyrrole ring. Interestingly, the active conformations of the unsubstituted hydrazides **11** and **12** were dissimilar (Figure 3A,B,E,F). The *p*-bromophenyl moiety of **11** was situated in the aromatic cage of the enzyme; however, the orientation of **12** shifted the available aromatic moiety and the unsubstituted hydrazide fragment in the aromatic cage. The latter established better in vitro MAO-B inhibitory activity of **12** compared to the hydrazide with shorted linker **11**. This could be explained by the deeper orientation of **12** in the active site of the enzyme and the smaller solvent-accessible area. The visualization of **12** demonstrated that the ethyl ester fragment is facing the entrance of the enzyme. Importantly, the bulky nature of compound **11l** led to several steric crashes in the active site of MAO-B and consequently unfavorable docking score. The indole fragment of the molecule was facing the aromatic cage, while the substituted pyrrole core was situated in the substrate cavity (Figure 3C,D).

### 3.2. In Vitro Assessment of Neurotoxicity and Neuroprotection of the Evaluated Derivatives

The direct or indirect effect of xenobiotics and natural biologically active structures that disrupt the nervous system of humans is known as neurotoxicity. This may be linked directly to the effect on the neuronal cells or with interference with metabolic processes on which the central nervous system (CNS) is extremely dependent [22].

Thus, the preliminary evaluation of the possible neurotoxicity of CNS-active synthetic substances is of high importance for their further consideration as biologically active pharmaceuticals.

The current literature indicates various evidence that oxidative damage and mitochondrial dysfunction participate actively in a cascade of processes causing degeneration of the dopaminergic neurons, thus leading to the development of parkinsonism [23,24,25,26,27].

Additional confirmation of the importance and the influence of oxidative stress on Parkinson’s disease (PD) development is the evaluation of the dopaminergic neuronal degeneration by modeling the motor aspects of PD with toxins causing oxidative stress such as 6-hydroxydopamine (6-OHDA), *tert*-butyl hydroperoxide (*t*-BuOOH) and iron ascorbate (Fe^2+^/AA), rotenone, 1-methyl-4-phenyl-1,2,3,6-tetrahydropyrirdine (MTPT), etc. [28,29,30].

This determined our aim to assess the toxic and neuroprotective effects of the hydrazones **11a**–**11x** of hydrazide **11** (ethyl 5-(4-bromophenyl)-1-(2-hydrazinyl-2-oxoethyl)-2-methyl-1*H*-pyrrole-3-carboxylate), and hydrazones **12a**–**12x** of hydrazide **12** (ethyl 5-(4-bromophenyl)-1-(3-hydrazinyl-3-oxopropyl)-2-methyl-1*H*-pyrrole-3-carboxylate).

The neurotoxic and neuroprotective effects of the target compounds were evaluated based on their influence on the subcellular viability, GSH and MDA levels. These experiments were based on the fact that the reduced glutathione (GSH) acts as a nucleophilic acceptor of a large number of compounds and their reactive (toxic) metabolites. It has been found that reducing the level of reduced glutathione by about 20–30% leads to a weakening of the cell’s defense mechanisms against toxic anions and can lead to cell damage and death. Thus GSH depletion is a well-applied indication of toxicity. Therefore, the amount of reduced glutathione is an important biomarker related to xenobiotic toxicity [31,32].

Another important marker of lipid peroxidation is malon aldehyde (MDA). It is a product of lipid peroxidation, obtained from the breakdown of hydroperoxides formed during the oxidation of polyunsaturated fatty acids. The most studied aldehydes formed in the process of lipid peroxidation are: 4-hydroxynonenal, 4-hydroxyhexenal and malon aldehyde [33]. MDA is a highly reactive metabolite and forms Schiff bases with the free amino groups of proteins and amino acids. It constitutes about 2% of lipid peroxidation products formed [34], diffuses easily and has a longer life than free radicals. Its increased production is a clear sign of toxicity.

There is a close relationship between the amount of GSH in the cell and the production of MDA. In most cases, the reduction of the GSH level caused by the action of the reactive (toxic) metabolite leads to the induction process of lipid peroxidation and increased MDA production.

The results from the performed evaluations determined most of the hydrazones to possess noticeable neurotoxicity, except for four representatives the two initial hydrazides **11** and **12** and the corresponding hydrazone **11l** of hydrazide **11** and **12a** of hydrazide **12**, related with the lowest toxicity, evaluated on three subcellular fractions, isolated from rat brain. As quantitative parameters in the synaptosomal fraction the corresponding synaptosomal viability and GSH depletion were applied (Figure 4 and Figure 5). For the mitochondrial fraction, the quantitative assessment was based on the measurement of the effects on the MDA production (Figure 6) and the GSH level effect (Figure 7). While in the microsomal fraction was of interest to identify the influence of the target compounds on the production of MDA (Figure 8). The most essential observation is the fact that on all evaluated parameters in all evaluated fractions the same four derivatives **11**, **12**, **11l** and **12a** were marketed with the least neurotoxicity.

In general, the neurotoxicity results identified that the hydrazones of hydrazide **12** (ethyl 5-(4-bromophenyl)-1-(3-hydrazinyl-3-oxopropyl)-2-methyl-1*H*-pyrrole-3-carboxylate) are with lower toxicity than the derivatives **11a**–**11x** of hydrazide **11** (ethyl 5-(4-bromophenyl)-1-(2-hydrazinyl-2-oxoethyl)-2-methyl-1*H*-pyrrole-3-carboxylate).

This determined their further assessment as possible neuroprotectors. These effects were evaluated on three available models of oxidative damage: 6-OHDA induced oxidative stress, *tert*-butyl hydroperoxide (*t*-BuOOH) induced oxidative stress, and non-enzymatic induced lipid peroxidation in the three isolated rat brain subcellular fractions. Again, as quantitative parameters were measured the effects on the synaptosomal viability and GSH depletion (Figure 9) in the synaptosomes; on the MDA production and GSH levels (Figure 10) in the mitochondria; and on the MDA production in the microsomes (Figure 11).

The results again extract as most perspective the hydrazides **11** and **12**, along with the hydrazones **11l** (ethyl 1-(2-(2-((1-acetyl-1*H*-indol-3-yl)methylene)hydrazinyl)-2-oxoethyl)-5-(4-bromophenyl)-2-methyl-1*H*-pyrrole-3-carboxylate) and **12a** (ethyl 1-(3-(2-benzylidenehydrazinyl)-3-oxopropyl)-5-(4-bromophenyl)-2-methyl-1*H*-pyrrole-3-carboxylate).

Interestingly, the obtained in vitro results indicated that the derivatives with a prolonged methylene bridge between the central pyrrole system and the heteroatomic hydrazone group display better neuroprotective properties.

In these evaluations, again, the availability of the free NH_2_ group in the hydrazides **11** and **12** determines the best manifestation of neuroprotection. These results are confirmed by extensive studies published, which indicated that molecules containing free R(Ar)(C=O)-NH-NH_2_ group are potent radical scavengers and antioxidants [35,36,37], thus indicating this group as an essential pharmacophore for such biological activity.

### 3.3. In Vitro Evaluation of the Targeted N-Pyrrolyl Hydrazide–Hydrazones on the Activity of Human Recombinant MAO-B Enzyme

The MAO-B inhibitory activities of all the targeted compounds were evaluated at 1 μmol and Selegiline (a MAO-B inhibitor) was selected as the reference drug. Several compounds exhibited significant MAO-B inhibitory effect at the applied concentration (Figure 12). In 1 μmol concentration, the pyrrole hydrazides **11** and **12** inhibited MAO-B with 26% and 50%, respectively. The hydrazide–hydrazone **12a**, which poses an additional benzene ring, showed inhibitory activity of 45%. These results could be related to the stabilization of the molecule due to interactions with the aromatic cage of the enzyme. The hydrazide–hydrazone with an indole fragment **11l**, demonstrated MAO-B blocking activity of 23%.

The difference in the in vitro MAO-B results for the two hydrazides could be explained by the dissimilar linker in **11** and **12**. In the case of **11**, glycine was applied as an amino acid to obtain the *N*-pyrrolyl carboxylic acid, while β-alanine was applied in **12**, thus inserting an additional methylene group in the bridge between the central pyrrole ring and the R(Ar)(C=O)-NH-NH_2_ group. This leads to the distancing of this group from the aromatic part of the structure, thus affecting its electronic properties.

The obtained in vitro results confirmed most of the docking simulations, which defined this method as suitable for future preliminary assessment of molecules based on the *N*-pyrrolyl hydrazide–hydrazone core. On the other hand, the low neurotoxicity, promising neuroprotection, and relatively good inhibitory MAO-B effects of the underlined derivative **12** may be a good starting point in the future development of promising agents affecting Parkinson’s conditions.

## 4. Materials and Methods

### 4.1. Chemistry

The evaluated compounds were synthesized through a classical Paal–Knorr condensation of a targeted 1,4-dicarbonyl compound with a previously selected amino acid for the formation of the basic pyrrole ring. The next steps include the esterification of the obtained *N*-pyrrolyl carboxylic acid, followed by a hydrazinolysis of the detached ester group to form the targeted hydrazides **11** and **12**. The last stage of the synthesis includes azomethine condensation of the resultant hydrazides with a series of carbonyl partners, as identified in Figure 1. A more detailed explanation of the synthesis of the compounds is available in [38] for hydrazide **11** and its hydrazones **11a**–**11x**, and in [39] for hydrazide **12** and its hydrazones **12a**–**12x**. The structures of the molecules were proven by the necessary IR, ^1^H-NMR and MS spectral data. Their purity was determined through proper TCL characteristics, melting points and elemental analyses, as described in [38,39].

### 4.2. Molecular Docking

#### 4.2.1. Hardware

The docking simulations were carried out on an AMD Ryzen 9 5950X 16 core CPU with GeForce RTX 3060 12 GB GPU, and 64 GB of installed RAM. The utilized operating system was 64 bit Windows 10 Pro.

#### 4.2.2. Selection and Preparation of Proteins

The crystallographic structures of MAO-B (PDB ID: 1S3B) resolved with the co-crystallized ligand *N*-[(1*S*)-2,3-dihydro-1*H*-inden-1-yl]-*N*-methyl-*N*-prop-2-ynylamine was retrieved from the Protein Data Bank (PDB). A recent study by our research group demonstrated that 1S3B possesses good reliability during validation processes [17]. The Protein Preparation Wizard in Maestro (Schrödinger Release 2021-3: Protein Preparation Wizard; Epik, Schrödinger, LLC, New York, NY, USA, 2021) was employed for the protein refinements. Hydrogen bonds and het states at pH 7.0 ± 2.0 were generated. The active waters were preserved. Subsequently, the energy of the crystallographic structures was minimized by applying the OPLS2005 force field.

#### 4.2.3. Preparation of Ligands

The chemical structures of the title pyrroles were drawn with the 2D sketcher module in Maestro and converted to the corresponding three-dimensional (3D) structure with the LigPrep module (Schrödinger Release 2021-3: LigPrep, Schrödinger, LLC, New York, NY, USA, 2021). Utilizing LigPrep, hydrogen bonds, tautomers and ionization states at pH 7.0 ± 2.0 were generated. Furthermore, the energies of the ligands were minimized by applying the OPLS2005 force field.

#### 4.2.4. Docking Protocol

The docking program GOLD (Genetic Optimization for Ligand Docking) was employed for the current docking study. GOLD 5.3 possesses four scoring functions—ChemPLP, GoldScore, ASP and Chemscore. The applied scoring function was ChemPLP, the grid space was set to 12 Å [40]. Molecular Mechanics/Generalized Born Surface Area (MM-GBSA) recalculations with Prime were also employed to assess the free binding energies of the obtained complexes. The calculations were performed by the incorporation of the OPLS3 force field and VSGB dissolvable model [41]. The grid box was generated around the obtained GOLD 5.3 docking poses.

### 4.3. Biological Evaluation

#### 4.3.1. Animals

The isolated subcellular fractions were obtained from a total of 10 old male Wistar rats (2 years of age). The animals were acquired from the National Breeding Center, Bulgarian Academy of Science, Slivnitza, Bulgaria, and were housed in standard laboratory conditions (ambient temperature 20 °C ± 2 °C and humidity 72% ± 4%) in plexiglass cages with a 12/12 h light/dark cycle and free access to water, according to ISO 9001:2008. Vivarium (certificate of registration of farm 0072/01.08.2007) was inspected by the Bulgarian Drug Agency to check the husbandry conditions (A-11-1081/03.11.2011). Twelve hours before each study, the animals were deprived of their food. All procedures were carried out in strict compliance with the requirements of the Institutional Committee for Animal Welfare and the principles set out in the European Convention for the Protection of Vertebrate Animals Used for Experimental and Other Scientific Purposes (ETS 123) (Council of Europe, 1991), throughout the experiment.

#### 4.3.2. Isolation and Incubation of Rat Brain Synaptosomes and Mitochondria

The synaptosomes and mitochondria were prepared through repeated differential centrifugation, using a Percoll gradient to separate the different fractions [42]. Both subcellular fractions were incubated with 100 µmol of the test substances.

#### 4.3.3. Model of 6-OHDA-Induced Neurotoxicity in Synaptosomes

This in vitro model resembles the neurodegenerative processes occurring in Parkinson’s disease (PD). Dopamine metabolism and oxidation lead to the formation of reactive oxygen species (ROS) and reactive quinones. They induce dopamine neurotoxicity and neurodegeneration [43]. The synaptosomes were incubated with 150 μmol 6-OHDA for 1 h [44].

#### 4.3.4. *Tert*-Butyl Hydroperoxide (*t*-BuOOH)-Induced Oxidative Stress in Isolated Brain Mitochondria

The mitochondria were incubated with 75 µmol *tert*-butyl hydroperoxide (*t-*BuOOH) and the tested compounds (100 µmol) for 1 h [45].

#### 4.3.5. Determination of Synaptosomal Viability and Reduced Glutathione (GSH) Level

The synaptosomal vitality was determined by the MTT method of Mungarro-Menchaca [46] with the GSH level evaluated by the Robyt method, as described in [47].

#### 4.3.6. Determination of Malondialdehyde (MDA) Production and Reduced GSH Level in Rat Brain Mitochondria

Both parameters were determined spectrophotometrically at wavelengths of 535 nm for MDA and 412 nm for GSH, respectively, as explained in Shirani et al. [48].

#### 4.3.7. Isolation of Rat Brain Microsomes 

The brain microsomes were prepared according to the procedure described in [49].

#### 4.3.8. Development of a Model of Non-Enzyme-Induced Lipid Peroxidation and Determination of MDA in Isolated Rat Brain Microsomes

The brain microsomes were pre-incubated with 40 µmol FeSO_4_ and 500 µmol ascorbic acid. The quantity of the lipid peroxidation product MDA was assessed spectrophotometrically at 535 nm. For the calculation of the MDA amount, a molar extinction coefficient of 1.56 × 10^5^ mol^−1^ cm^−1^ was used [50].

#### 4.3.9. Evaluation of Human Monoamine Oxidase B (*h*MAOB) Activity

The monoamine oxidase type B activity was assayed on recombinant human MAOB using a fluorimetric method applying Amplex^®^ UltraRed reagent [51] with small modifications [52] where tyramine hydrochloride was used as substrate and Selegiline as a positive control.

### 4.4. Statistical Analysis

The results obtained on isolated rat brain synaptosomes, mitochondria and microsomes were statistically processed with the statistical program ‘MEDCALC’, using the non-parametric Mann–Whitney method, and on *h*MAOB parametric Student’s test for paired and group data at statistical significance *p* < 0.05.

## 5. Conclusions

A group of *N*-pyrrolyl hydrazide–hydrazones were evaluated through in silico and in vitro approaches for performing neurotoxic, neuroprotective and MAO-B inhibitory effects. The evaluations identified four representatives as favorable agents, with **12** underlined as most promising. The in vitro results confirmed most of the docking simulations, which defined this method as suitable for future preliminary assessment of molecules based on the *N*-pyrrolyl hydrazide–hydrazone core. On the other hand, the low neurotoxicity, promising neuroprotection, and relatively good inhibitory MAO-B effects of the underlined derivative **12** may be a good starting point in the future development of perspective agents affecting Parkinson’s conditions.

## Figures and Tables

**Table 1:** GOLD 5.3 and MM/GBSA recalculations of pyrrole-based compounds in the crystallographic MAO-B structure (PDB ID: 1S3B).

	Docked Compounds									
	12a	12	11c	11	11d	11n	11b	11l	Selegiline	Safinamide
ChemPLP fitness score	124.38	132.97	129.15	134.40	109.22	123.85	115.70	96.88	140.28	166.40
MM/GBSA	−56.21	−54.46	−46.44	−44.15	−41.04	−33.85	−12.31	57.59	−50.80	−79.72

**Table 2:** Active MAO-B amino residues participated in the interactions with **11**, **11l**, **12**, **12a**, Selegiline and Safinamide.

Compound	Hydrophobic Interaction	Polar Interaction	Hydrogen Bond	π–π Interaction	Steric Clashes
**11**	Tyr60, Pro102, Trp119, Leu164, Leu167, Phe168, Leu171, Cys172, Tyr188, Ile198, Ile199, Ile316, Leu328, Met341, Phe343, Tyr398, Trp432, Tyr435	Gln206	-	Tyr326	-
**11l**	Tyr60, Pro102, Trp119, Leu164, Leu167, Phe168, Leu171, Cys172, Val173, Tyr188, Ile198, Ile199, Ile316, Leu328, Met341, Phe343, Tyr398, Trp432, Tyr435	His115, Ser200, Thr201, Gln206, Thr314	Tyr188	Tyr398	Glu84, Leu164, Ile199
**12**	Tyr60, Pro102, Phe103, Pro104, Trp119, Leu164, Leu167, Phe168, Leu171, Cys172, Val173, Tyr188, Ile198, Ile199, Ile316, Leu328, Met341, Phe343, Tyr398, Trp432, Tyr435	Gln206	-	Tyr326	-
**12a**	Tyr60, Pro102, Phe103, Pro104, Trp119, Leu164, Leu167, Phe168, Leu171, Cys172, Tyr188, Ile198, Ile199, Ile316, Leu328, Met341, Phe343, Tyr398, Tyr435	Gln206	-	Try326, Tyr398	-
**Selegiline**	Tyr60, Phe168, Ile199, Gln206, Leu328, Met341, Phe343, Tyr326, Tyr398, Tyr435	Gln206	FAD	Leu171, Cys172, Ile198	-
**Safinamide**	Tyr60, Phe103, Pro104, Leu171, Ile199, Gln206, Phe343, Tyr398, Tyr435	Gln206	Ile199, FAD	Leu171, Ile199, Ile316, Tyr326	-

## Data Availability

Not applicable.

## Sample Availability

Samples of the compounds **11**, **11a**–**11x**,**12**, **12a**–**12x** are available from the authors.
